# Multidisciplinary management of interstitial lung disease in autoimmune rheumatic diseases: an Italian Delphi consensus

**DOI:** 10.1186/s12931-026-03660-z

**Published:** 2026-04-11

**Authors:** Marco Sebastiani, Elena Bargagli, Stefania Cerri, Serena Guiducci, Andreina Manfredi, Giacomo Sgalla, Carlo Vancheri, Elisabetta Zanatta, Fabrizio Luppi

**Affiliations:** 1 Rheumatology Unit, AUSL Piacenza, Piacenza, Italy; 2 Department of Medicine and Surgery, University of Parma, Parma, Italy; 3 Respiratory Diseases Unit, Department of Medical Surgical Sciences and Neurosciences, University Hospital of Siena, Siena, Italy; 4 Respiratory Disease Unit, Center for Rare Lung Disease, University Hospital of Modena, Modena, Italy; 5 Division of Rheumatology, Department of Experimental and Clinical Medicine, University of Florence, Careggi Hospital, Florence, Italy; 6 S.C. Rheumatology, University of Modena and Reggio Emilia, USL-IRCCS Reggio Emilia Health Authority, Reggio Emilia, Italy; 7 Unità Operativa Complessa di Pneumologia, Fondazione Policlinico Universitario “A. Gemelli” IRCCS, Università Cattolica del Sacro Cuore, Rome, Italy; 8 Department of Clinical and Experimental Medicine, University - Hospital Policlinico “G. Rodolico-San Marco”, University of Catania, Catania, Italy; 9 Rheumatology Unit, Department of Medicine, University of Padova, Padova, Italy; 10 Department of Cardiac, Thoracic, Vascular Sciences and Public Health, University of Padova, Padova, Italy; 11 Pneumology Unit, Fondazione IRCCS San Gerardo dei Tintori, Monza, Italy; 12 Department of Medicine and Surgery, University of Milano Bicocca, Monza, Italy

**Keywords:** Delphi consensus, Interstitial lung disease, Multidisciplinary team, Progressive pulmonary fibrosis, Rheumatoid lung disease

## Abstract

**Background:**

Although guidelines recommend a multidisciplinary team (MDT) approach for the diagnosis and management of systemic autoimmune rheumatic disease-associated interstitial lung disease (SARD-ILD), they lack guidance on MDT composition and function. This Delphi consensus project aimed to define a shared MDT model for managing patients with SARD-ILD.

**Methods:**

A questionnaire was circulated to an expert panel of 77 Italian pulmonologists, rheumatologists, immunologists, and internal medicine specialists, with statements rated over two voting rounds using a 5-point Likert scale.

**Results:**

Response rates were 73% and 95% for the first and second rounds, respectively, after which consensus (≥ 66.6% agreement) was achieved for all the statements. Consensus statements and recommendations address the responsibilities of healthcare professionals involved in an MDT, including organization, referrals, management, and standard outcomes.

**Conclusion:**

Although limited to Italy, the current consensus project is the first attempt to define shared rules for MDTs in the context of SARD-ILD, but further work is needed to achieve international consensus on this topic.

**Supplementary Information:**

The online version contains supplementary material available at 10.1186/s12931-026-03660-z.

## Background

Interstitial lung disease (ILD) comprises a broad group of several lung disorders that vary according to etiology [1–5]. ILD can be caused by a variety of factors, and the types of ILD can be grouped based on their underlying causes and histopathological features [4, 6]. Among them, systemic autoimmune rheumatic disease-associated ILD (SARD-ILD), comprising approximately 20% of all ILDs, is associated with autoimmune diseases such as rheumatoid arthritis (RA), primary Sjogren’s syndrome (pSS), systemic sclerosis (SSc), polymyositis, and dermatomyositis [7–10]. SARD-ILD can result in fibrosis and diffuse damage to the lungs, which can progressively impair lung function [4–6, 11–16]. ILD largely affects morbidity and mortality, representing a leading cause of death in patients with autoimmune diseases [4, 11, 12, 17, 18].

Epidemiological data regarding SARD-ILD are inconsistent, making the prevalence uncertain [1, 5, 10, 11]. However, it is estimated that up to 64% of patients with SSc and at least 10% of patients with RA and pSS can develop ILD [1, 5, 7–12]. Also, the prevalence is thought to be increasing over time, likely reflecting both an ageing population and improved diagnosis [5].

Treatment of SARD-ILD is aimed at delaying disease progression with immunosuppressive therapy and antifibrotic agents, and management of symptoms (e.g., exercise tolerance) with supplemental oxygen and pulmonary rehabilitation [3, 6, 11, 17, 19–22]. Given the heterogeneity of ILD [5], a multidisciplinary team (MDT) approach is currently considered the gold standard for diagnosis [2, 4, 23, 24]. Evaluation of disease severity, ILD progression and response to treatment is also best performed by an MDT [4, 11, 12, 24]. Cooperation between the various specialized healthcare practitioners (HCPs) in an MDT is known to provide improved treatment efficiency and patient care [24, 25]. Furthermore, a broad spectrum of extrapulmonary manifestations must be considered in the management of patients with SARD-ILD [23, 26].

Despite guideline recommendations for the use of MDTs for managing patients with SARD-ILD, no guidance is provided on the composition and function of MDTs, which can vary between centers and countries [23]. Proposed by a group of expert Italian pulmonologists and rheumatologists, this Delphi consensus study aimed to identify and propose an efficient organizational model of an MDT specifically for the management of patients with SARD-ILD.

## Methods

### Study design

The current study used Delphi methodology to develop consensus recommendations based on insights from expert physicians regarding the diagnosis, treatment, and management of patients with SARD-ILD using a multidisciplinary approach. The Scientific Advisory Board consisted of nine members (the authors), who facilitated group communication with a wider panel of experts to reach consensus on a set of statements following two rounds of voting and a final (third) meeting to discuss the overall results [27]. Members of the Expert Panel (pulmonologists, rheumatologists, immunologists, and internal medicine specialists who had known experience in SARD-ILD) were chosen from referral centers in Italy that utilize an MDT approach to the management of ILD.

### Delphi questionnaire

A Scientific Advisory Board, comprising five pulmonologists and four rheumatologists from across Italy (authors of this article), was convened to develop a consensus definition regarding the role of an MDT for the management of patients with SARD-ILD. The topics examined included the composition, aims, and organization of an MDT. The statements were developed based on available published evidence alongside the clinical experience of the Scientific Advisory Board.

### Search strategy and selection criteria

A literature search was conducted in Medline/PubMed, EMBASE and Scopus for articles published up to September 2023, using the terms (“interstitial lung disease” OR “pulmonary fibrosis”) AND (“connective tissue diseases” OR “collagen vascular diseases” OR “autoimmune rheumatic diseases” OR “rheumatic diseases” OR “rheumatoid arthritis” OR “systemic sclerosis” OR “scleroderma” OR “Sjogren syndrome” OR “systemic vasculitis” OR “inflammatory myopathies” OR “anti-synthetase syndrome”) AND (“multidisciplinary team” OR “multidisciplinary discussion” OR “multidisciplinary evaluation”). Editorials, conference abstracts, case stories, smaller case series, and pre-print publications were excluded.

The preliminary statements were then discussed at an online meeting of the Scientific Advisory Board members held on December 20, 2023, and a second meeting was held on February 1, 2024, to conclude discussions and finalize the Delphi questionnaire statements. At the second meeting, criteria for choosing an Expert Panel to vote on the statements were also defined. The initial Delphi questionnaire comprised 49 statements (seven related to the roles of the various HCPs involved with an MDT; seven related to the organization of an MDT for ILD; 16 related to the referral and management of cases; and 19 related to standard outputs and deliverables of an MDT; Table S1 in the Supporting Information).

### Delphi voting

For the first round of Delphi voting, the questionnaire contained spaces for the Expert Panel to provide comments. The results obtained after the first round of voting were discussed in an online meeting held on July 11, 2024. All questions were re-evaluated, comments from the Expert Panel were discussed, and the questionnaire modified prior to circulating to the Expert Panel for the second round of voting. The modified questionnaire was approved by the Scientific Advisory Board and distributed for the second vote.

During both rounds of voting, members of the Expert Panel rated their agreement/disagreement with each of the proposed statements using a 5-point Likert scale (1 = strongly disagree, 2 = disagree, 3 = neither agree nor disagree, 4 = agree, 5 = strongly agree). Similar to other Delphi studies on respiratory diseases [28–30], consensus was considered to have been reached if ≥ 66.6% of the Experts responded that they “strongly agree” or “agree” with a statement. Following compilation of the results of the questionnaire, a final meeting was with the Scientific Advisory Board held online on November 6, 2024, to allow critical discussion of the results.

## Results

### Expert panel

Members of the Expert Panel evaluated the statements by means of their scientific and clinical expertise. Of the 77 experts initially approached for inclusion in the study, 56 (73%) responded to the first Delphi questionnaire. Among these, stated specialties were rheumatology (*n* = 27), pulmonology (*n* = 26), immunology (*n* = 2), and internal medicine (*n* = 1).

### Results of the first round of Delphi votes

High consensus in responses was achieved at the first Delphi vote; 48/49 statements achieved consensus and only one statement (Statement 30) did not reach consensus (58.9%; Table S1 in the Supporting Information). Statement 30 was considered problematic because it completely excluded patients previously diagnosed in other centers with SARD-ILD that is stable over time, whereas it was thought that these patients could be included but were not a priority for MDT discussion. Twenty-six statements were modified, mainly for minor grammatical changes, and based on comments provided by panelists. Statements 26 and 45 were deleted because they were considered repetitive of information in other statements. Statement 46 was designed to obtain criteria that should be used for the validation of MDTs. Thus, the Expert Panel’s comments were used to formulate seven new statements in the new version of the questionnaire, comprising 52 statements in total.

### Results of the second round of Delphi votes

Round 2 of the Delphi voting was completed by 53 of the 56 (95%) members of the Expert Panel (Table 1). Consensus was reached for all 52 statements.

**Table 1:** Statements 1–52 from the final round of the Delphi process

	Statement	Level of agreement (*N* = 53)
1. Roles of the various HCPs involved with an MDT		
*1a. Core HCPs*		
1	The core HCPs in an MDT for SARD-ILD should ideally include a pulmonologist, a thoracic radiologist, and a rheumatologist	100.0%
*1b. Added value of an MDT*		
2	Treatment decisions made by an MDT come from a collaborative effort, considering concurrently both pulmonary and extra-pulmonary aspects of the disease	98.2%
3	The MDT approach influences management strategies by facilitating a more coordinated and integrated care model, which can lead to early diagnosis, optimized treatment plans, and potentially better outcomes than traditional care models	98.1%
4	Regarding patients with SARD-ILD that are managed by both a pulmonologist and a rheumatologist, and according to specific case-by-case needs, the MDT should be responsible for, not only of the diagnosis of new cases, but also for therapeutic decision-making and follow-up of ongoing cases	83.0%
*1c. Other specialists occasionally included in the MDT*		
5	Considering the systemic nature of rheumatic diseases, other specialists might be included in the MDT according to the specific organ involvement in each patient	88.7%
6	In cases of suspected or confirmed PH, an expert in PH should be involved in the MDT in the context of rheumatic diseases	98.1%
*1d. Role of PCPs in the MDT*		
7	PCPs are useful liaisons for an MDT, ensuring continuity of care for patients with ILD. Their role includes initial patient screening, referral to specialists, and regular updates on treatment progress	73.6%
2. Organization of an MDT for ILD		
*2a. Structure of a standard agenda*		
8	The agenda for MDT meetings should include (and formally acknowledge) adequate time for physicians to present and discuss findings	100.0%
9	MDT meetings should concentrate on making and reviewing the diagnosis, as well as defining optimal treatment options and follow-up outcomes in accordance with the referring physician	98.1%
10	Follow-up strategies for each patient should be clearly defined and assigned to respective team members	98.1%*
*2b. Frequency of MDT meetings*		
11	MDTs should meet regularly according to the number of cases to be discussed. Generally, a meeting should be planned at least every 2 weeks to ensure prompt discussion and management of cases	84.9%
12	In centers with a high volume of patients, more frequent meetings may be necessary to handle the caseload effectively	90.6%
*2c. Supportive operating structures*		
13	In selected cases, when the therapeutic choice is going to affect both pulmonary and extra-pulmonary involvement, or when direct clinical evaluation is needed, a joint evaluation, including both pulmonologist and rheumatologist, could be proposed	86.6%*
14	For its effectiveness, the MDT should be able to rely on electronic medical records for real-time data access. To facilitate access to the MDT, a cloud-based system for efficient data storage and virtual collaboration among physicians is advisable	86.6%*
3. Referral and management of cases		
*3a. Referral of cases for discussion by the MDT*		
15	As a standard procedure, it is the responsibility of any treating physician to refer cases of ILD to an MDT	100.0%
16	When cases are proposed by other colleagues, their active involvement in the MDT is advisable	86.8%
*3b. Preparation of cases for discussion by the MDT*		
17	Cases for MDT discussion are typically prepared and presented by the referring specialist, often a pulmonologist or rheumatologist, who provides a comprehensive summary of the patient’s history, current clinical status, and diagnostic findings to facilitate effective decision-making during the meeting	100.0%
18	It is useful for the effectiveness of the MDT to have a case manager in charge of scheduling MDT meetings, follow-up visits, and analysis of patient outcomes	96.2%
19	The clinical query to the MDT should be clearly stated in the patient case file	96.3%
20	It is important to use a standardized template for collecting medical history data	77.3%
21	For rheumatology patients, all extrapulmonary manifestations/comorbidities that may affect prognosis should be reported	100.0%
*3c. Patient management*		
22	Transition of care for patients referred to or from other specialties should be managed by ensuring clear communication among all involved parties and establishing a well-defined care pathway for each patient	100.0%
23	An MDT approach to management of patients with ILD should be aligned with national healthcare policies and guidelines, focusing on early intervention strategies	100.0%*
24	MDTs should regularly review and analyze outcomes of cases in order to identify areas for improvement in the quality of patient care and the functionality of the MDT	94.4%
25	Preliminary MDT consultations should be restricted to professionals, in the absence of the patient, with the feedback subsequently returned to the patient. However, MDT members may, in some cases, choose to meet together with the patient, who thus receives direct communication	90.4%*
26	Patients with ILD, regardless of treatment, who develop new symptoms suggestive of autoimmune rheumatic diseases should be discussed by the MDT to confirm the diagnosis, and to evaluate the need for a change in treatment and follow-up strategy. A rheumatologic assessment before the MDT meeting is also advisable	92.5%
27	Patients with a definite autoimmune rheumatic disease with new evidence of ILD may not require an MDT to confirm diagnosis; however, an MDT can be useful to stage ILD severity and to evaluate the need for treatment	88.7%
28	Patients previously diagnosed with SARD-ILD that is stable over time without the need for treatment cannot be prioritized in MDT discussions	79.2%
4. Standard outputs and deliverables of an MDT		
*4a. Essential features of MDT reports*		
29	An ILD MDT report should summarize patient status, detail specialist contributions, and outline a consensus-based treatment plan. Expected outputs of MDT meetings include diagnosis, tailored treatment plans, follow-up schedules, and management strategies for comorbid conditions	92.4%
30	The MDT output must clearly stick to the clinical query and state the diagnosis (first hypothesis and possible secondary diagnoses)	94.3%
31	The MDT output must clearly stick to the clinical query and state evaluation of disease progression	100.0%
32	The MDT output must clearly stick to the clinical query and state the therapeutic approach	100.0%
33	The MDT output must clearly stick to the clinical query and state the need for re-evaluation of the patient	92.3%*
34	The MDT output must clearly stick to the clinical query and state instructions for follow-up (including timing)	92.5%
35	Outputs of an MDT should be communicated and implemented in patient care through documented meeting minutes, formal communication channels with all involved HCPs, and direct patient engagement	79.3%
36	An MDT report should be easily accessible to all relevant HCPs, ensuring continuity of care and adherence to the agreed-upon treatment strategy	94.4%
37	An MDT should adapt its approach based on progression or response to treatment of ILD by reassessing treatment efficacy, considering alternative therapies, and adjusting management plans accordingly	72.1%*
38	Strategies for quality assurance and improvement of MDT outputs include regular reviews of treatment outcomes, patient feedback mechanisms, and adherence to updated clinical guidelines	92.3%*
*4b. Supporting infrastructure and facilities*		
39	The hospital where the MDT is located must offer an adequate number of available specialists	96.2%*
40	The hospital where the MDT is located must offer the availability of appropriate platforms/services for sharing diagnostic data and imaging	94.4%
41	The hospital where the MDT is located must offer the availability of second- and third-level equipment to perform rheumatological, pulmonological, and radiological assessments	98.1%
42	The hospital where the MDT is located must offer specific training programs in ILD for HCPs	90.6%
*4c. Features of a validated MDT*		
43	An MDT should be composed of physicians with expertise in diagnosis and management of patients with ILD according to national and international guidelines	98.1%
44	A validated MDT for the management of patients with ILD should meet according to a regular schedule. The meetings should follow a standardized case referral template, and track outcomes (time to diagnosis, revised diagnoses, patients lost to follow-up, progression of ILD) and the drugs prescribed	94.3%
45	A validated MDT for the management of patients with ILD should ideally include a clinical case manager who ensures prompt diagnostic work-up, patient feedback, and follow-up	94.4%
46	A validated MDT for the management of patients with ILD should have expertise in managing SARD-ILD, meet minimum case numbers, engage in continuous education, and have endorsement as a referral center	98.1%
47	A validated MDT for the management of patients with ILD should include specialists (at least a pulmonologist, radiologist, and rheumatologist) with specific clinical experience. Participation in studies, clinical trials, and other research activities should be valued	96.3%
48	A validated MDT for the management of patients with ILD should provide team members with effective information sharing, adequate technological resources, and case management	100.0%
49	A validated MDT for the management of patients with ILD should include dedicated ILD outpatient clinics, shared pulmonologist–rheumatologist clinics, a hub-and-spoke network, together with a high-level autoimmunity laboratory and radiology unit	96.2%
50	To ensure that MDT members maintain qualifications, ongoing education programs, participation in conferences, and regular training sessions must be implemented	100.0%
51	Training of MDT members should focus on improving patient-centered communication, with specific attention to patient needs and awareness of the latest national and international guidelines for the management of ILD	98.1%
52	The performance of an MDT should be evaluated based on metrics such as patient outcomes, adherence to treatment guidelines, and patient satisfaction scores	79.2%

*HCPs* healthcare practitioners, *ILD* Interstitial lung disease, *MDT* Multidisciplinary team, *PCPs* Primary care physicians, *PH* Pulmonary hypertension, *SARD-ILD* Systemic autoimmune rheumatic disease-associated interstitial lung disease

*One expert did not respond to this statement (i.e., *n* = 52)

## Discussion

Consensus statements from each topic are discussed below, along with supporting evidence from the literature.

When considering the roles of the various HCPs in an MDT for the management of patients with SARD-ILD, the Scientific Advisory Board sought consensus from the Experts on core HCPs and the added value of an MDT, other specialists who might provide occasional input to an MDT, and how primary care physicians (PCPs) might interact with an MDT.

Regarding Statement 1 (Table 1), which focuses on the core HCPs in an MDT, previous international guidelines [31–33] and consensus recommendations have stated that an ILD MDT should consist of a pulmonologist, a thoracic radiologist, and a pathologist [23, 34, 35]. Despite proven improvements in diagnostic accuracy and clinical outcomes when included in an MDT, rheumatologists are not routinely involved in ILD MDTs in Italy [24]. However, in cases of suspected SARD-ILD, it has been suggested that an MDT should also include a rheumatologist [36]. Furthermore, it has been shown that the presence of a rheumatologist in the MDT has a substantial impact in modifying the diagnosis and consequent therapies of referred patients [24, 37]. Additionally, in patients with primarily rheumatological pathologies, joint evaluation with a pulmonologist can increase the diagnostic sensitivity of ILD.

When considering the added value of an MDT over and above that offered by the individual HCPs (Statements 2–4, Table 1), it is important to consider the role of an MDT across all stages of the patient management pathway. While use of an MDT approach has been shown to facilitate early and accurate diagnoses [38], in the Italian setting, an MDT should also be involved in therapeutic decision-making.

Regarding Statements 5 and 6, which consider other specialists occasionally included in the MDT (Table 1), in Italy, the Scientific Advisory Board noted that pathologists are not usually involved in the discussion of SARD-ILD cases because, in accordance with the latest American and European Guidelines for the diagnosis and management of SARD-ILD [12, 39], most patients do not undergo a biopsy. However, when a biopsy is available, their involvement in the MDT meeting becomes indispensable, as the accurate interpretation of histopathological data plays a key role in diagnosing ILD [31, 32]. Thereafter, longitudinal management is mainly driven by clinical phenotype, pulmonary function trends, and treatment response, roles primarily undertaken by pulmonologists and rheumatologists once diagnosis is established. Additionally, due to the systemic nature of autoimmune rheumatic diseases, other specialists – such as cardiologists, nurses, and allied health professionals (psychologists, physiotherapists, and nutritionists) – may be included as a part of the MDT based on the specific organ involved or local practices. For example, in Italy, the management of pulmonary hypertension may require the expertise of either a cardiologist or a pulmonologist, depending on the clinical context.

While PCPs are not typically included in MDTs, the interface and continuity in care that they provide for patients with SARD-ILD ideally makes them useful points of contact for an MDT (Statement 7, Table 1) [40]. Belief among the Scientific Advisory Board members was that PCPs in Italy are unlikely to attend MDT meetings if invited. Thus, the role of PCPs includes initial patient screening, referral to specialists, regular updates on treatment progress, and management of comorbidities. Indeed, PCPs are pivotal in coordinating long-term care and patient education. Therefore, in order to highlight the crucial role that MDTs play in the management of patients with SARD-ILD, comprehensive training and education of PCPs is essential [36, 40].

When considering the organizational structure of an MDT for the management of patients with ILD (Statements 8–10, Table 1), the Scientific Advisory Board sought consensus from the Experts on the structure and frequency of MDT meetings, and other organizational structures, platforms, and information support that might be necessary. The structure of a standard agenda of an ILD MDT is related to the objectives of the MDT (i.e., evaluating new cases, generating a differential diagnosis, reviewing ongoing cases, and discussion of treatment options).

The setting for ILD MDT meetings should enable easy interaction between attendees (Statements 11 and 12, Table 1) [35]. Studies conducted in other countries have shown that MDT meetings are generally held weekly or fortnightly and are of 31–90 min duration [34, 35]. However, the frequency of MDT meetings will depend on the number of cases of ILD managed by the center.

Since referring physicians are responsible for sharing all relevant patient medical records (e.g., medical history, clinical notes, radiological imaging, histopathology reports) when referring a patient with suspected SARD-ILD to an MDT [35, 36], access to electronic medical records is crucial (Statements 13 and 14, Table 1). Additionally, efficient data systems for secure storage and seamless sharing of patient information between specialists and PCPs are essential.

When considering the referral and management of patients to an MDT (Statements 15 and 16, Table 1), the Scientific Advisory Board sought consensus from the Experts regarding the referral and preparation of cases for discussion by an MDT, as well as how patients with ILD should be managed by an MDT.

Early referral is essential, since delayed referral is associated with a higher risk of death in patients with ILD [36]. Early identification of patients at risk of progressive pulmonary fibrosis may lead to earlier interventions that could result in improved outcomes [41], e.g., patients with early-stage ILD may benefit from lifestyle interventions, such as smoking cessation and occupational or environmental risk reduction [41]. It has been shown that transplant-free survival was significantly reduced when lung function (as measured by forced vital capacity) had decreased by ≥ 10% [42]. Thus, early identification and careful follow-up may allow prompt identification of patients to be treated. Moreover, diagnostic delay of ILD is a predictive factor of mortality in SARD-ILD [43], with the suggestion that increased mortality related to diagnostic delay may be due to delayed MDT evaluation resulting in inferior treatment management. Similarly, a previous United States-based study suggested that patients with diagnostic or treatment uncertainty, disease progression despite treatment, and transplant or clinical trial candidates would benefit from specialist referral [36].

Streamlining MDT meetings improves decision-making efficiency for routine cases requiring standard care, allowing more time for focused discussions on clinically challenging cases (Statements 17–21, Table 1) [44].

Pre-meeting preparation of all cases (simple and complex) by a specialist should include making summaries of all relevant information from electronic notes, and of radiology and histological results from hospital data systems [44].

Management of systemic and lung involvement in patients with SARD-ILD generally requires the use of multiple therapeutic strategies, since treatment of joint involvement does not appear to affect lung disease; immunosuppressive drugs that are effective in lung disease do not improve arthritis (Statements 22–28, Table 1) [8]. Also, the potential for pulmonary toxicity with conventional synthetic and biological disease-modifying antirheumatic drugs [18, 45] adds further complexity to the treatment of RA-related ILD [8]. Moreover, treatment of patients with systemic sclerosis and idiopathic inflammatory myopathies requires consideration of several other frequent clinical manifestations, such as skin and muscle involvement or cardiac involvement [26, 46].

Typically, the referral specialist should be responsible for sharing any decisions made by the MDT with the patient (Statements 29–38, Table 1). Thus, when considering standard outputs and deliverables of an MDT for ILD, the Scientific Advisory Board sought consensus from the Expert Panel regarding essential features of MDT reports that might help this communication, plus the requirements for supporting infrastructure and/or facilities and essential features of a validated MDT.

Given their importance for coordinating patient management, MDT reports should accurately document meeting proceedings and provide details of subsequent treatment and care for patients [47]. Moreover, completeness, accessibility and usability of MDT reports are essential [47]. Electronic records are believed to provide improved legibility, completeness and organization of information, as well as provide decision support [47]. Real-time electronic notetaking during the meeting is considered optimal, providing efficiency and removal of duplication of work.

The importance of supporting infrastructure and facilities is covered by Statements 39–42 (Table 1), wherein centers must have the necessary number of physicians skilled in the diagnosis and treatment of patients, plus the availability of appropriate technology to support them to function effectively, all of which is paramount to the success of ILD MDTs [23, 35].

Validation/certification of MDTs helps ensure that patients receive the best evidence-based care (Statements 43–52, Table 1) [48]. Measurement of the quality of MDTs requires definition of characteristics of effectiveness to provide a framework against which to develop objective assessment criteria [48]. Effective MDT functioning is impacted by the quality of the composition and skills of the team, the supportive infrastructure, meeting organization and team governance, and patient-centered clinical decision-making and treatment protocols [48, 49]. While preferable features of an effective MDT process have been established in the care of other diseases [49, 50], these are currently lacking for SARD-ILD.

### Limitations

A potential limitation of this study is that, since Delphi consensus studies rely on expert opinion, their content and applicability is limited by the experts included, as well as any inherent bias introduced by their non-random selection [51, 52]. Further, although mentioned, no pathologists or thoracic radiologists were included. The composition of our Expert Panel was designed to align with our primary objective, which was to harmonize clinical management strategies for SARD-ILD, with a focus on identification of progression, monitoring, and treatment decisions. In addition, our study lacked input from the patient perspective in the development of the consensus statements. We recommend that future panels include other specialists (pathologists, thoracic radiologists, cardiologists), nurses, allied health professionals (psychologists, physiotherapists, nutritionists), and patient representatives.

## Conclusions

This Delphi review demonstrated consensus among Italian experts on the essential and desirable features of an MDT for the diagnosis and management of patients with SARD-ILD. More work is needed to ensure effective functioning of ILD MDTs in Italy and their future standardization.

## Supplementary Information

Supplementary Material 1.

## Data Availability

The data that support the findings of this study are available from the corresponding author upon reasonable request.

## Abbreviations

HCPs: Healthcare practitioners
ILD: Interstitial lung disease
MDT: Multidisciplinary team
PCPs: Primary care physicians
PH: Pulmonary hypertension
pSS: Primary Sjogren’s syndrome
RA: Rheumatoid arthritis
SARD-ILD: Systemic autoimmune rheumatic disease-associated interstitial lung disease
SSc: Systemic sclerosis
