# Supplementary material for: Multidisciplinary management of interstitial lung disease in autoimmune rheumatic diseases: an Italian Delphi consensus
Source: Respir Res. 2026 Apr 11;27:228. doi: 10.1186/s12931-026-03660-z (PMC13262451; doi:10.1186/s12931-026-03660-z)
Supplement: Supplementary file 1 — Supplementary Material 1. [file 12931_2026_3660_MOESM1_ESM.docx]

# SUPPORTING INFORMATION

**MULTIDISCIPLINARY MANAGEMENT OF INTERSTITIAL LUNG DISEASE IN AUTOIMMUNE RHEUMATIC DISEASES: AN ITALIAN DELPHI CONSENSUS**

**Authors:** Marco Sebastiani, Elena Bargagli, Stefania Cerri, Serena Guiducci, Andreina Manfredi, Giacomo Sgalla, Carlo Vancheri, Elisabetta Zanatta, Fabrizio Luppi

**Corresponding author:** Marco Sebastiani; Email: marco.sebastiani@unipr.it. ORCiD ID: 0000-0002-1294-6421

## Table S1.

Statements 1–49 from the first round of the Delphi process.

| **No.** | **Statement** | **Level of agreement (N=56)** |  |
| --- | --- | --- | --- |
| **1. Roles of the various HCPs involved with an MDT** | | | |
| ***1a. Core HCPs*** | | | |
| 1 | The core HCPs in an MDT for ILD should ideally include a pulmonologist, pathologist, thoracic radiologist, and rheumatologist | 98.2% |  |
| ***1b. Added value of an MDT*** | | | |
| 2 | Treatment decisions made by the MDT come from a collaborative effort, considering concurrently both pulmonary and extra-pulmonary aspects of the disease | 100.0% |  |
| 3 | The MDT approach influences management strategies by facilitating a more coordinated and integrated care model, which can lead to early diagnosis, optimised treatment plans, and potentially better outcomes compared with traditional care models | 100.0% |  |
| 4 | Regarding patients with ILD that are managed by both a pulmonologist and a rheumatologist, and according to specific case-by-case needs, the MDT should be in charge not only of the diagnosis of new cases but also for the therapeutic decision and of the follow-up of ongoing cases | 94.7% |  |
| ***1c. Other specialists occasionally included in the MDT*** | | | |
| 5 | Considering the systemic nature of rheumatic diseases, other specialists might be included in the MDT according to the specific organ involvement in each patient | 81.5%* |  |
| 6 | The cardiologist should be part of the MDT in the context of rheumatic diseases with suspected PH | 76.4%^‡^ |  |
| ***1d. Role of PCPs in the MDT*** | | | |
| 7 | PCPs are useful liaisons for ILD MDTs, ensuring continuity of care. Their role includes initial patient screening, referral to specialists, and regular updates on treatment progress | 75.0% |  |
| **2. Organisation of an MDT for ILD** | | | |
| ***2a. Structure of a standard agenda*** | | | |
| 8 | The agenda for MDT meetings should include and formally acknowledge adequate time for physicians to present and discuss findings | 98.2% |  |
| 9 | MDT meetings should concentrate on making and reviewing the diagnosis | 83.9% |  |
| 10 | Follow-up strategies for each patient should also be clearly defined and assigned to respective team members | 94.7% |  |
| ***2b. Frequency of MDT meetings*** | | | |
| 11 | MDTs should meet regularly according to the number of cases to be discussed. Generally, a meeting should be planned at least every 2 weeks to ensure prompt discussion and management of cases | 80.1%^‡^ |  |
| 12 | In centres with a high volume of patients, more frequent meetings may be necessary to handle the caseload effectively | 91.1% |  |
| ***2c. Supportive operating structures*** | | | |
| 13 | In selected cases, when the therapeutic choice is going to affect both pulmonary and extra-pulmonary involvement, or when direct clinical evaluation is needed, a joint evaluation, including both pulmonologist and rheumatologist, could be proposed | 89.3% |  |
| 14 | For its effectiveness, MDT should be able to rely on electronic medical records for real-time data access. To facilitate access to the MDT, a cloud-based system for efficient data storage and virtual collaboration among physicians is advisable | 94.6%^‡^ |  |
| **3. Referral and management of cases** | | | |
| ***3a. Referral of cases for discussion by the MDT*** | | | |
| 15 | As a standard procedure, it is the responsibility of any treating physician to refer cases of ILD to the MDT | 89.1^‡^ |  |
| 16 | When cases are proposed by other colleagues, their active involvement in the MDT is advisable | 94.6% |  |
| ***3b. Preparation of cases for discussion by the MDT*** | | | |
| 17 | Cases for MDT discussion are typically prepared and presented by the referring specialist, often a pulmonologist or rheumatologist, who provides a comprehensive summary of the patient’s history, current clinical status, and diagnostic findings to facilitate effective decision-making during the meetings | 100.0% |  |
| ***3c. Inclusion of a case manager*** | | | |
| 18 | It is useful for the effectiveness of the MDT to have a case manager in charge of scheduling MDT meetings, follow-up visits, and analysis of patient outcomes | 85.7% |  |
| 19 | The clinical query to the MDT should be clearly stated in the patient's file | 96.4% |  |
| 20 | It is important to use standardised templates for collecting medical history data | 78.6% |  |
| 21 | For rheumatology patients, all extrapulmonary manifestations/comorbidities that may affect prognosis should be reported | 98.2% |  |
| ***3e. Patient management*** | | | |
| 22 | Transition of care for patients referred to or from other specialties is managed by ensuring clear communication among all involved parties and establishing a well-defined care pathway for each patient | 94.6% |  |
| 23 | An MDT's approach to ILD should be aligned with national healthcare policies and guidelines, focusing on early intervention strategies | 100.0% |  |
| 24 | An MDT adapts its approach based on the progression or response to treatment of ILD by reassessing treatment efficacy, considering alternative therapies, and adjusting management plans accordingly | 98.3% |  |
| 25 | An MDT should regularly review and analyse the outcomes of managed cases to identify areas for improvement in the quality of patient care and the functionality of the MDT | 94.6% |  |
| 26 | Preliminary consultations should be restricted to professionals, in the absence of the patient, with the feedback subsequently returned to the patient. Still, MDT members may in some cases choose to meet together with the patient, who thus receives live communications | 71.4% |  |
| 27 | Patients previously diagnosed with idiopathic ILD, regardless of the treatment, who develop symptoms suggestive of autoimmune rheumatic diseases should be discussed by the MDT to confirm the diagnosis, and to evaluate the need for a change in treatment and follow-up strategy | 98.2% |  |
| 28 | Patients with stable, well-defined SARD-ILD who develop new respiratory symptoms or require new treatment for ILD and/or treatment for potentially life-threatening autoimmune rheumatic disease should be evaluated by the MDT | 92.8%^‡^ |  |
| 29 | Patients with a definite autoimmune rheumatic disease with new evidence of ILD usually don’t require MDT to confirm diagnosis; however, MDT can be useful to stage ILD severity and to evaluate the need for treatment | 75.0% |  |
| 30 | New patients previously diagnosed with SARD-ILD that is stable over time without need for treatment cannot be discussed by an MDT | 58.9% |  |
| **4. Standard outputs and deliverables of an MDT** | | | |
| ***4a. Essential features of MDT reports*** | | | |
| 31 | An ILD MDT report should summarize patient status, detail specialist contributions, and outline a consensus-based treatment plan. Expected outputs of MDT meetings include diagnosis, tailored treatment plans, follow-up schedules, and management strategies for comorbid conditions | 94.5% |  |
| 32 | The MDT output must clearly stick to the clinical query and state the diagnosis (first hypothesis and possible secondary diagnoses) | 98.2% |  |
| 33 | The MDT output must clearly stick to the clinical query and state evaluation of disease progression | 96.4% |  |
| 34 | The MDT output must clearly stick to the clinical query and state the therapeutic approach | 96.4%^‡^ |  |
| 35 | The MDT output must clearly stick to the clinical query and state the need for re-evaluation of the patient | 91.1% |  |
| 36 | The MDT output must clearly stick to the clinical query and state instructions for follow-up (including timing) | 96.4% |  |
| 37 | These outputs are communicated and implemented in patient care through documented meeting minutes, formal communication channels with all involved HCPs, and direct patient engagement | 87.3%^‡^ |  |
| 38 | The report should be easily accessible to all relevant HCPs, ensuring continuity of care and adherence to the agreed-upon treatment strategy | 92.9% |  |
| 39 | Strategies for quality assurance and improvement of MDT outputs include regular reviews of treatment outcomes, patient feedback mechanisms, and adherence to updated clinical guidelines | 94.4%* |  |
| ***4b. Supporting infrastructure and facilities*** | | | |
| 40 | The hospital where the MDT is located must always be capable of an adequate number of available specialists | 98.2% |  |
| 41 | The hospital where the MDT is located must always be capable of the availability of appropriate platforms/services for sharing diagnostic data and imaging | 94.6% |  |
| 42 | The hospital where the MDT is located must always be capable of the availability of second- and third-level equipment to perform rheumatological, pulmonological, and radiological assessment | 96.4% |  |
| 43 | The hospital where the MDT is located must always be capable of specific training programmes in ILD for HCPs | 76.8% |  |
| ***4c. Features of a validated MDT*** | | | |
| 44 | An MDT should be composed of physicians with expertise in diagnosis and management of patients with ILD according to national and international guidelines, which should provide the references to identify the minimum level of expertise for a professional to be included in an MDT | 87.3%^‡^ |  |
| 45 | ILD MDTs should ideally comply to a set of requirements and be validated according to the parameters therein | 87.3%^‡^ |  |
| 46 | Please list here the criteria that you would recommend so as to generate a benchmark to be used for the validation of MDTs in ILD | (various answers)^§^ |  |
| 47 | To ensure that MDT members maintain necessary qualifications, ongoing education programs, participation in conferences, and regular training sessions must be implemented | 96.5% |  |
| 48 | Training of MDT members should focus on improving patient-centred communication, with specific attention to patient needs, and awareness of the latest national and international guidelines for the management of ILD | 92.9% |  |
| 49 | The performance of the MDT should be evaluated based on metrics such as patient outcomes, adherence to treatment guidelines, and patient satisfaction scores | 85.7% |  |

*Two experts did not respond to this statement (i.e., n=54).

‡One expert did not respond to this statement (i.e., n=55).

§21 experts did not provide a response to this statement (i.e., n=35).

HCPs, healthcare practitioners; ILD, interstitial lung disease; MDT, multidisciplinary team; PCPs, primary care providers; PH, pulmonary hypertension; SARD-ILD, systemic autoimmune rheumatic disease-associated interstitial lung disease.
